# Commentary: Plaque Features and Epicardial Fat Volume for Cardiovascular Risk Assessment—A Key Role With Cardiac Computed Tomography?

**DOI:** 10.3389/fcvm.2022.896321

**Published:** 2022-04-29

**Authors:** Christian Tesche, Alexander Giesen, Grigorios Korosoglou

**Affiliations:** ^1^Department of Cardiology, Clinic Augustinum Munich, Munich, Germany; ^2^Department of Cardiology, Munich University Clinic, Ludwig-Maximilians-University, Munich, Germany; ^3^Division of Cardiovascular Imaging, Department of Radiology and Radiological Science, Medical University of South Carolina, Charleston, SC, United States; ^4^Department of Cardiology, Vascular Medicine and Pneumology, Gesundheitszentrum Rhein-Neckar Hospital Weinheim, Weinheim, Germany; ^5^Cardiac Imaging Center Weinheim, Hector Foundation, Weinheim, Germany

**Keywords:** epicardial fat tissue, pericoronary adipose tissue attenuation, low-attenuation plaque, high-risk plaque features, cardiac outcomes, troponin

Over the last 2 decades, coronary CT angiography (CCTA) has emerged as a cornerstone with a class I recommendation for the assessment of coronary artery disease (CAD) (1–3). CCTA allows for combined anatomical and morphological, and if required functional assessment of CAD. In addition, the multicentric, randomized SCOT Heart trial previously demonstrated a significantly lower rate of cardiovascular death or non-fatal myocardial infarction at 5 years of follow-up, in patients who received a primary CCTA vs. standard care strategy, with similar rates of coronary revascularization procedures in both arms (4). In the same direction, a subsection analysis of this trial demonstrated that low attenuation, non-calcified plaque burden is the most robust predictor of future fatal and non-fatal myocardial infarction, beyond calcium score and coronary artery stenosis (5). In addition, a recently published multicentric, randomized trial further strengthens the applicability of a primary CCTA strategy in patients with chronic coronary syndromes, since it resulted in significant lower procedure-related complications such as myocardial infarction and stroke with simultaneously similar rates of major adverse cardiovascular events during long-term follow-up, compared to a primary invasive strategy (6).

Detailed plaque quantification and characterization with the identification of so called “high-risk” plaque features like low-attenuation plaque (LAP) or the “Napkin ring sign” as markers of vulnerable plaques has demonstrated incremental value to identify patients at risk for future cardiovascular events (7, 8). A growing body of evidence supports the value of epicardial adipose tissue (EAT) assessment as a surrogate marker of coronary artery inflammation in addition to the evaluation of coronary plaque extent and morphology (9). Thus, previous studies showed that EAT quantification is closely associated with atherosclerotic plaque burden and the presence of obstructive CAD (10). In addition, EAT recently exhibited incremental value for the prediction of future cardiovascular events compared to conventional risk score models, thus representing a valuable imaging biomarker for the risk stratification of such patients (11).

In the current issue of *Frontiers in Cardiovascular Medicine—Cardiovascular Imaging* Otsuka and colleagues (12) retrospectively assessed the ability of CCTA-derived low-attenuation non-calcified plaque burden to predict cardiovascular outcomes. In addition, Otsuka et al. investigated the impact of epicardial adipose tissue volume (EAV) on low-attenuation plaque burden in the same patient cohort. Overall, 376 symptomatic patients without known CAD (57% male, 65 ± 13 yrs. old, 22% with diabetes mellitus) with clinical indication for CCTA were analyzed. Most patients were not on cardiac medications, including lipid-lowering therapy and platelet inhibitions at the time of the baseline CCTA (only 5 and 26% on treatment with aspirin and statins, respectively). EAV and coronary plaque burden were assessed using commercially available software tools. All quantitative measures exhibited reasonable reproducibility.

Among the 376 patients included in the trial, the primary endpoint was observed in 15 patients during a mean follow-up of 2.2 ± 0.9 yrs., including death in 2, acute coronary syndromes in 6 and urgent revascularization in seven patients, respectively. Low-attenuation plaque burden was highly predictive for future cardiac events, independent of traditional CAD risk scores, coronary calcification, coronary stenosis severity and EAV (HR 3.05, 95%CI 1.09–8.54, *p* = 0.033 by adjusted Cox regression analysis). Thus, most patients who reached the primary endpoint, were in the highest low-attenuation plaque burden quartile (Q4). EAV on the other hand, was not predictive for future clinical endpoints but was associated with low-attenuation plaque burden (*r* = 0.386, *p* < 0.001). Thus, both EAV ≥125 ml and calcium score ≥218.3 were associated with the presence of the highest low-attenuation plaque burden quartile (Q4).

The report of the study by Otsuka et al. (12) needs to be considered in the context of the current cardiovascular risk assessment approaches. Despite continuous advances in the diagnosis of CHD, sudden death and acute ischemic syndromes continue to be leading causes of morbidity and mortality (13), whereas almost 50% of patients with a sudden cardiac death does not experience limiting clinical symptoms before (14). The results presented by Otsuka et al. agree with previous reports, demonstrating the value of coronary plaque burden and composition for the prediction of future cardiovascular events (5, 10, 15). In addition, the association of EAV with low-attenuation plaque burden could be nicely shown. EAT is the fat tissue located between the visceral pericardium and the myocardium and is widely recognized having numerous exocrine and paracrine effects by the secretion of bioactive substances called adipokines. Both EAT as a whole and EAT within a radial distance from the outer wall of coronary vessels, the so called pericoronary adipose tissue (PCAT) (16), exhibit complex bidirectional interactions with the underlying vascular wall. Thus, dysfunctional EAT and PCAT are involved in the production of proinflammatory adipokines, which may cause the activation of inflammatory pathways *via* paracrine or vasocrine effects, resulting in endothelial dysfunction, increase coagulability and vasoconstriction, smooth muscle cell proliferation and ultimately atherogenesis progression (17). Importantly, EAT and PCAT inflammation were shown to be associated with CAD progression and adverse cardiovascular events in several clinical studies (18–21). Importantly, in contrast to other imaging modalities, such as echocardiography and cardiac magnetic resonance (CMR), CCTA can visualize and precisely quantify both EAT, PCAT and coronary plaque composition and burden within a single examination, as illustrated in Figure 1 (9).

**Figure 1:**
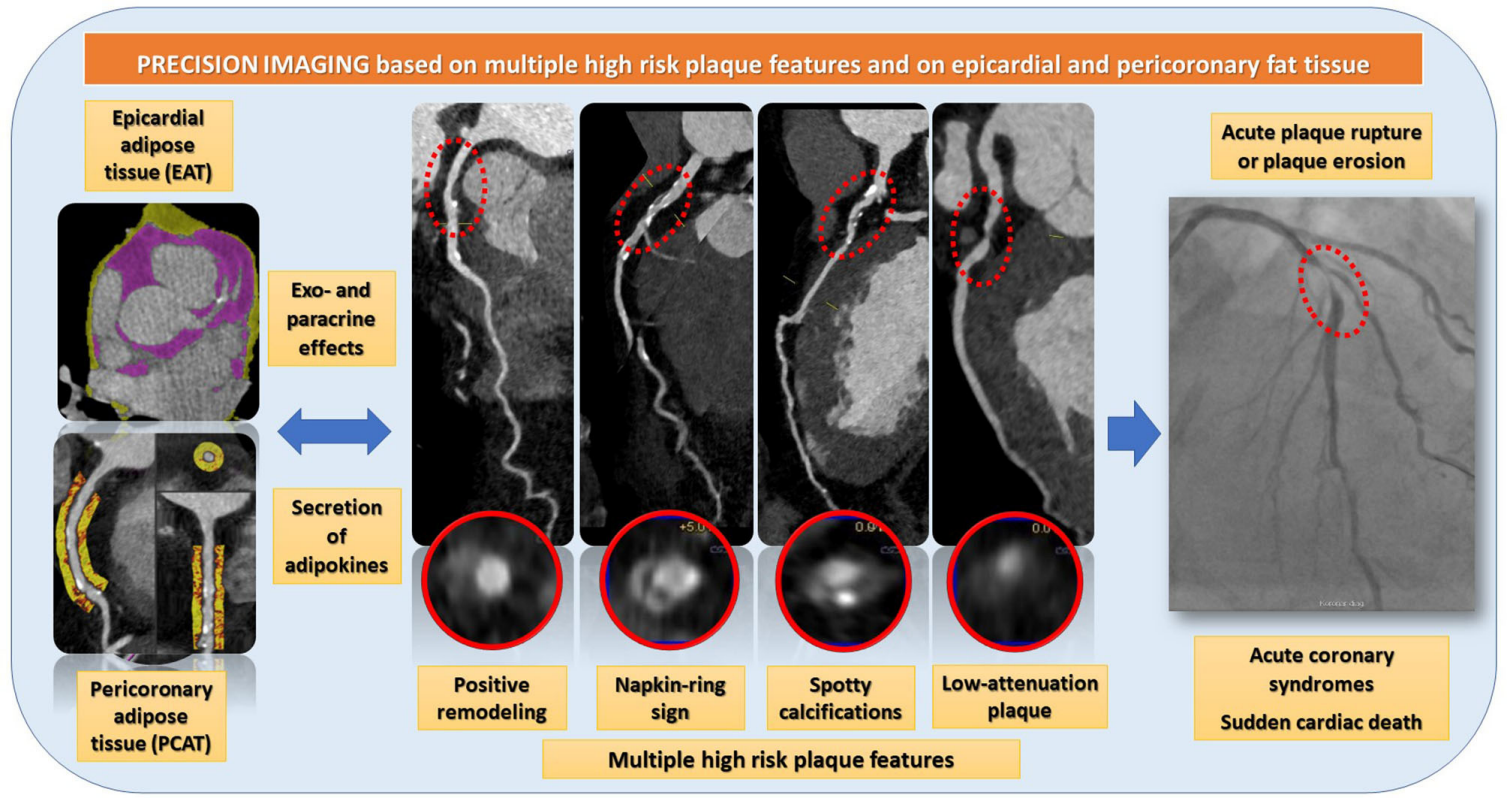
CCTA can visualize and precisely quantify both EAT, PCAT and coronary plaque composition and burden within a single examination. EAT and PCAT are associated with high-risk plaque features, such as positive remodeling, the napkin ring sign, spotty calcifications, and low-attenuation plaques. Such high-risk plaques are rupture prone and therefor potential precursors of acute coronary syndromes.

Some limitations need to be considered when interpreting the results of the present study. From a technical point of view, the assessment of plaque burden and EAV was not purely automatic, so that manual corrections were necessary when using semiautomated plaque and EAV quantification algorithms. This represents a time-consuming process, hampering the translation of such quantification approaches in the clinical realm. This limitation, however, may be overcome by the implementation of artificial intelligence and machine-learning algorithms for fully automated atherosclerotic plaque quantification and characterization derived from CCTA data together with incorporation of “big data” from electronic health records for personalized decision-making and risk prediction (22). Notably, the authors only included low attenuation plaque burden as a central marker of in their analysis, whereas other high risk plaque features, such as the “napkin-ring sign,” spotty calcifications, and the positive vessel remodeling index were not considered. Such a ‘multi-feature' approach may improve the predictive value of CCTA and may be the way to go in future studies. In addition, biochemical markers, such as cardiac troponins, which were previously shown to be associated with plaque composition and cardiovascular outcomes (23, 24) were not analyzed in the present study, which is a limitation. Furthermore, the study was performed retrospectively, and the number of cardiac events was relatively small, especially when focusing on hard cardiac events, such as death and myocardial infarction. Thus, larger studies are warranted to further elucidate the relationship between EAT, PCAT and multiple high risk plaque features for the prediction of cardiovascular outcomes.

Despite these limitations, the study by Otsuka et al. (12) provides excellent evidence that EAV is associated with low-attenuation plaque burden, which in turn is the most robust variable for the prediction of future adverse cardiovascular events. In the era of precision medicine, quantification assessment of such parameters may help identifying patients at high risk for future cardiovascular events, tailoring therapeutic and preventions strategies in such individuals. Health care systems will also definitely need to consider adapting reimbursement strategies in this context, providing incentives for the broader use of CCTA over the much more costly invasive procedures in individuals who develop cardiovascular complications.

## Disclosure

CT receives honoraria for speaking and consulting from HeartFlow Inc. and Siemens Healthineers. GK receives institutional research grants from Siemens Healthineers.

## Author Contributions

All authors listed have made a substantial, direct, and intellectual contribution to the work and approved it for publication.

## Conflict of Interest

The authors declare that the research was conducted in the absence of any commercial or financial relationships that could be construed as a potential conflict of interest.

## Publisher's Note

All claims expressed in this article are solely those of the authors and do not necessarily represent those of their affiliated organizations, or those of the publisher, the editors and the reviewers. Any product that may be evaluated in this article, or claim that may be made by its manufacturer, is not guaranteed or endorsed by the publisher.
